# Etiological Factors and Visual Outcomes of Dense Vitreous Hemorrhage in Patients Aged 80 years and above over the Past Decade in a Tertiary General Hospital

**DOI:** 10.1155/2023/8851207

**Published:** 2023-09-28

**Authors:** Yuhua Ding, Bangtao Yao, Hui Ye, Fei Wang

**Affiliations:** ^1^Department of Ophthalmology, Jiangsu Province Hospital, The First Affiliated Hospital of Nanjing Medical University, Nanjing, Jiangsu, China; ^2^Department of Ophthalmology, Nanjing Lishui People's Hospital, Zhongda Hospital Lishui Branch, Southeast University, Nanjing, Jiangsu, China

## Abstract

This study aimed to investigate the main etiological factors and visual outcomes in patients with dense vitreous hemorrhage (DVH) aged ≥80 years. We retrospectively included patients with DVH aged ≥80 years who were admitted to our ophthalmology department between January 1, 2010, and December 31, 2019. All patients underwent pars plana vitrectomy (PPV). Data regarding demographic characteristics; preoperative and postoperative best-corrected visual acuity (BCVA), intraocular pressure (IOP), and ophthalmic B-scan ultrasonography findings; intraoperative conditions; and postoperative complications were collected and analyzed. A total of 44 patients (44 eyes) were enrolled, with a median age of 82 years; among them, 25 patients (56.82%) were men. The median preoperative BCVA was 2.3 (1.1–3.0). The main etiological factors included retinal vein occlusion (RVO) (20 eyes, 45.45%), polypoidal choroidal vasculopathy (PCV) (15 eyes, 34.09%), proliferative diabetic retinopathy (PDR) (7 eyes, 15.90%), retinal arterial macroaneurysm (RAM) (1 eye, 2.27%), and posterior vitreous detachment (PVD) (1 eye, 2.27%). The median final BCVA was 1.92 (0.5–2.6). There was a significant postoperative improvement in the BCVA; moreover, branch RVO (BRVO) had a better postoperative visual prognosis than central RVO (CRVO), PCV, and PDR (*P* < 0.05). The final postoperative BCVA was significantly better when the initial BCVA was above hand motion (HM) than when it was HM or lower (*P* < 0.05). Our findings indicate that RVO, PCV, and PDR were the main causes of DVH. Microinvasive PPV is a safe and effective method that can clarify diagnosis and improve BCVA. Patients with BRVO and preoperative BCVA > HM may have a relatively good visual prognosis. For patients aged ≥80 years who have an appropriate general condition, PPV can be timely performed to treat DVH.

## 1. Introduction

Population aging is a global problem. Worldwide, the proportion of people aged ≥65 years is expected to increase from 6.9% in 2000 to 16.4% in 2050, while the proportion of very elderly individuals is expected to increase from 1.9% to 4.2% [1]. Although the very elderly population accounts for a small proportion of the human population, it comprises a significant proportion of ophthalmic patients.

Vitreous hemorrhage (VH) is among the most common ocular diseases and is known to cause vision loss. Dense VH (DVH) in patients aged ≥80 years seriously affects vision, which increases the risk of falling and fracture, and thus increases the burden on the family and affects the survival rate [2, 3]. Accordingly, DVH is an increasingly concerning problem among patients aged ≥80 years.

In clinical settings, identifying the cause of DVH is often challenging because the fundus is not easily visible. Furthermore, the clinical causes of DVH may differ between older and younger patients. In addition, the characteristics and prognosis of DVH in elderly patients with DVH, especially those aged ≥80 years, remain unclear. Therefore, this study aimed to investigate the main etiological factors and visual outcomes of DVH in patients aged ≥80 years.

## 2. Materials and Methods

### 2.1. Study Participants

We retrospectively reviewed the electronic medical records of patients with DVH aged ≥80 years who were admitted to the Department of Ophthalmology, Jiangsu Province Hospital, between January 1, 2010, and December 31, 2019. This study adhered to the tenets of the Declaration of Helsinki. Furthermore, this study was approved by the Ethics Committee of Jiangsu Province Hospital.

The diagnostic criteria for DVH were as follows: (1) best-corrected visual acuity (BCVA) ≤0.1; (2) severe VH, which was too dense to allow visualization of the posterior pole of the retina; and (3) B-scan ultrasound showing no signs of retinal tear or retinal detachment (RD). The exclusion criteria were as follows: a history of VH due to retinal vein occlusion (RVO), posterior vitreous detachment (PVD), recent trauma, Terson's syndrome, and so on, which had been absorbed without intervention when they were younger than 80 years old; had a history of glaucoma; having previously undergone pars plana vitrectomy (PPV); a diagnosis of advanced dementia; and a follow-up period of <6 months.

We collected demographic data including age and sex. The data regarding clinical characteristics, including intraoperative findings; complications; and follow-up duration, were also collected. The patients were examined before surgery as well as 1 day; 1 week; and 1, 3, and 6 months after surgery. The patients underwent comprehensive ophthalmic examinations, including BCVA measurement (E standard logarithmic visual acuity chart), intraocular pressure (IOP) measurement, slit-lamp dilated biomicroscopy, and indirect ophthalmoscopy. Optical coherence tomography (OCT), fundus fluorescein angiography (FFA), and indocyanine angiography (ICGA) were performed postoperatively in some patients.

### 2.2. Surgical Procedure

All surgeries were performed by experienced vitreoretinal specialists. After retrobulbar block using a 4 ml mixture of 0.75% bupivacaine and 2% lidocaine in a 1 : 1 ratio, a standard three-port 23-gauge, or 25-gauge vitrectomy with a wide-angle, noncontact viewing system (Resight®; Carl Zeiss Meditec AG, Jena, Germany) was performed using the constellation vision system (Alcon Laboratories Inc., Fort Worth, TX, USA). For phakic eyes, combined surgery involving phacoemulsification and intraocular lens implantation was performed using the same machine before vitrectomy. During vitrectomy, thick vitreous blood around the vitreous cutting head and optical fiber was initially removed. Subsequently, the anterior and posterior vitreous cavities were removed. If necessary, triamcinolone acetonide was injected to mark the residual vitreous cortex. Using assisted scleral indentation, clear and meticulous shaving of the vitreous base was performed using a wide-angle viewing system. Next, the organized vitreoretinal bands as well as proliferative and neovascular membranes were carefully separated and resected. Hemostasis was achieved by raising the IOP or using endodiathermy. Laser photocoagulation was performed for ischemic areas or holes. At the end of surgery, a balanced salt solution, air, or silicone oil was used to fill the vitreous cavity, as appropriate.

### 2.3. Statistical Analysis

Statistical analyses were performed using SPSS software (version 23.0; SPSS, Inc., Chicago, IL, USA). Continuous data are presented as medians and ranges. Categorical data are presented as absolute counts and percentages. BCVA values were converted into the logarithm of the minimal angle of resolution (logMAR) for statistical analysis [4]. The resulting logMAR equivalents were employed for low BCVA (0.3 = 0.5, 0.25 = 0.6, 0.2 = 0.7, 0.15 = 0.8, 0.12 = 0.9, 0.1 = 1.0, 0.08 = 1.1, 0.04 = 1.4, 0.02 = 1.7), counting fingers (CF; at 40 cm = 1.85, at 30 cm = 1.9, and at 10 cm = 2.0), hand motions (HM; 2.3), light perception (LP; 2.6), and no light perception (3.0). The Mann–Whitney *U* test and Kruskal–Wallis test were used for between-group and among-group comparisons, respectively. Statistical significance was set at *P* < 0.05.

## 3. Results

This study included 44 eyes in 44 patients (18 right and 26 left eyes; 25 males and 19 females). The median age of the patients was 82 (80–89) years. Confirmed by PPV intraoperative findings, as well as postoperative OCT, FFA, and ICGA findings, the most common etiological factors of DVH were RVO (20 eyes, 45.45%), polypoidal choroidal vasculopathy (PCV; 15 eyes, 34.09%), proliferative diabetic retinopathy (PDR; seven eyes, 15.90%), retinal macroaneurysm (RM; one eye, 2.27%), and PVD (one eye, 2.27%). The median ages of patients with DVH caused by RVO, PCV, PDR, RM, and PVD were 81.0, 82.4, 81.0, 85.0, and 82.0 years, respectively. Among patients with RVO, branch RVO (BRVO) was the most common subetiology (Table 1).

The BCVA improved, decreased, and was unchanged in 40 (90.91%), 1 (2.27%), and 3 (6.82%) patients, respectively. The median preoperative and final BCVA values were 2.3 (1.1–3.0) and 1.92 (0.5–2.6), respectively, which indicated a significant postoperative improvement (*P* < 0.05) (Table 2).

The patients were divided into four groups according to the four main causes. There was no significant among-group difference in postoperative BCVA (Table 3). However, the BRVO group had significantly better postoperative visual prognosis than the other three groups (Table 4).

In addition, patients were divided into two groups based on the initial BCVA as follows: group A, BCVA ≤ HM, and group B, BCVA > HM. Group B showed a significantly better final postoperative BCVA than group A (Table 5).

The IOP of all patients was within the normal range before and after surgery. No serious complications such as suprachoroidal hemorrhage or recurrent RD occurred.

## 4. Discussion

VH occurs secondary to various ocular or systemic diseases. A small amount of vitreous blood can be absorbed without intervention; however, vitreous blood in VH is difficult to absorb, which seriously affects the patient's vision and impedes diagnosis by ophthalmologists. Specifically, extensive unabsorbed vitreous blood may cause proliferative reactions in the vitreous, form abnormal neovascular fibrous proliferative membranes, and cause massive hemorrhage. Furthermore, the proliferative membrane can pull the retina to form holes or even lead to RD. Therefore, we routinely perform PPV to treat DVH and to clarify its etiological factors.

PDR, retinal vasculitis, and BRVO have been reported as the major causes of VH in adults [5, 6]. Given the unique physiological characteristics of the very elderly population, they may have different etiological factors of DVH. However, the specific characteristics and prognosis of very elderly patients with DVH, particularly those aged ≥80 years, remain unclear. We found that the most common etiologies in these patients were RVO, PCV, and PDR, which is inconsistent with previous reports [5, 6]. The disease spectrum and treatment strategies of the elderly group over 80 years old are different from those of middle-aged people. The elderly population is a vulnerable group and often even gives up treatment without intervention. Therefore, we aimed to investigate the causes of vitreous hemorrhage in elderly patients over 80 years old and whether treatment is meaningful.

In our study, the main cause of DVH in patients aged ≥80 years was RVO. DVH is a severe advanced complication of RVO, which is caused by dilation of obstructed blood vessels and rupture of retinal neovascularization. Hypertension is the strongest systemic risk factor for RVO [7]. Since the central retinal veins and arteries are located in the same adventitial sheath within the lamina cribrosa, arterial stiffness may affect the adjacent veins, resulting in CRVO [8]. BRVO may result from vein compression at arteriovenous crossings, degenerative changes within the venous walls, and hypercoagulability [8]. Systolic blood pressure is positively correlated with age, which could be the main factor for the increase in the incidence and prevalence of hypertension with age [9].

The second most common cause of DVH in our study was PCV. PCV is characterized by persistent, recurrent serous leakage, and hemorrhage in the macula in elderly individuals, which is caused by a peculiar form of choroidal neovascularization in the inner choroid [10]. Characteristic abnormalities observed in PCV include a branching vascular network and polypoidal structures at the lesion boundary. Asian ethnicity is associated with the risk of developing PCV [11, 12]. Tsujikawa et al. reported that a relatively large PCV (lesion area ≥1 optic disc area) was associated with an increased risk of extensive subretinal hemorrhage [13], which is more likely to cause dense breakthrough VH [14]. Patients with PCV and hypertension, diabetes, and cardiovascular disease have an increased risk of recurrent subretinal bleeding; moreover, these systemic diseases are very common in the very elderly population. Compared with classic age-related macular degeneration, hemorrhagic type PCV presents as hemorrhagic pigment epithelial detachment, with more subretinal hemorrhage in the macula and more frequent occurrence of explosive severe vitreous hemorrhage, resulting in a sudden decrease in the patient's vision. Furthermore, the limited number of enrolled patients was also an influencing factor. Thus, in this study, classic age-related macular degeneration patients were not collected.

The third most common cause of DVH in our study was PDR. PDR results from retinal ischemia and is characterized by the growth of new blood vessels on the surface of the retina or optic disc. These abnormal vessels may lead to VH, subsequent fibrosis, and tractional RD. The incidence of diabetic retinopathy (DR) increases with age and clinical progression of diabetes [15]. However, at the individual level, diabetes causes greater life loss and has a higher premature death index than hypertension [16].

We observed a significant postoperative improvement in BCV (*P* < 0.05). Notably, one patient with PCV showed vision improvement in the early postoperative period; however, she suffered from retinal rehemorrhage and her vision eventually deteriorated. The patient refused further treatment with antivascular endothelial growth factor (anti-VEGF). Among the remaining patients, the BCVA improved and remained unchanged in 40 and 3 patients, respectively. PPV can clear cloudy vitreous and inflammatory factors in the vitreous body to facilitate identification of the conditions of the retina and choroid. Laser treatment was administered intraoperatively as supplementary treatment, if necessary.

Among the etiologies, BRVO had a better postoperative visual prognosis than the other three etiologies (*P* < 0.05).

The visual prognosis of RVO is related to retinal blood supply, macular edema, and optic nerve conditions. Compared with BRVO, CRVO involved worse visual prognosis, which could be primarily attributed to widespread ischemic retinal damage, severe macular edema, or glaucomatous optic nerve damage.

Submacular hemorrhage is a serious complication of PCV that can lead to severe and irreversible damage to the photoreceptors and outer nuclear layer [17]. In the PCV group, all patients had submacular hemorrhage and, as a result, had a poor visual prognosis. Other common macular manifestations of severe PCV are associated with poor BCVA. For example, persistent macular serous RD causes the degeneration and atrophy of the macular retinal pigmentary epithelium; moreover, subretinal fibrovascular proliferation seriously impairs macular function [18]. However, most patients with PCV describe subjective improvement due to an improved visual field.

In our study, the PDR group had a worse visual prognosis than the BRVO group. Elderly patients with diabetes and DVH usually have poor control of blood sugar and blood pressure. The poor visual prognosis in this subgroup could be attributed to chronic high glucose levels and ischemia in the retina and optic nerve.

In our study, we found that the final postoperative BCVA was significantly better when the initial BCVA was above the HM than when the BCVA was HM or lower (*P* < 0.05). This indicates that preoperative visual function is positively correlated with the postoperative visual prognosis.

This study has several strengths. First, the data were collected from a tertiary general hospital. Compared with specialized hospitals, general hospitals have stronger comprehensive medical strength and a larger geriatric ward; moreover, they have more elderly patients, which is conducive to this study. Second, the observation period (10 years) allowed extensive collection of useful information. Moreover, within the past 10 years, there has been rapid development of minimally invasive PPV technology; accordingly, more patients aged ≥80 years with DVH may have had the opportunity for surgery. Therefore, the results of this study are representative.

However, this study had several limitations. First, given the recent reforms to China's medical insurance system, an increasing number of patients can afford antiVEGF treatment. However, among our patients, few patients could afford multiple anti-VEGF treatments owing to high costs. Second, as a new technology, intravitreal injection of a recombinant tissue plasminogen activator has been used to treat patients with PCV who develop extensive macular hemorrhage at an early stage, which can reduce the toxic effect of blood on the retinal pigmentary epithelium and photoreceptors [19]. Although these interventions can improve the vision and prognosis of patients, they were not administered to our patients. Third, the present study is a retrospective design.

## 5. Conclusions

In conclusion, RVO, PCV, and PDR are the main causes of DVH in patients aged ≥80 years. Microinvasive PPV is a safe and effective method for facilitating diagnosis and improving VA. Patients with BRVO and preoperative BCVA > HM may have a relatively good visual prognosis. For patients aged ≥80 years who have an appropriate general condition, PPV can be timely performed to treat DVH.

## Figures and Tables

**Table 1:** Etiology of DVH in patients aged ≥80 years.

Diagnosis	*N* (%)	Median age	Min. age	Max. age
RVO	20 (45.45)	81.0	80.0	84.0
BRVO	14			
CRVO	6			
PCV	15 (34.09)	82.4	80.0	89.0
PDR	7 (15.90)	81.0	80.0	86.0
RM	1 (2.27)	85.0		
PVD	1 (2.27)	82.0		

RVO: retinal vein occlusion; BRVO: branch retinal vein occlusion; CRVO: central retinal vein occlusion; PCV: polypoidal choroidal vasculopathy; RAM: retinal arterial macroaneurysm; *N*: number of eyes.

**Table 2:** Comparison of BCVA (logMAR) before and after surgery.

BCVA	Median	Range	*P* value
Initial	2.3	1.1–3.0	0.001
Final	1.92	0.5–2.6	

BCVA: best-corrected visual acuity.

**Table 3:** Comparison of preoperative BCVA among the four major etiological groups.

BCVA	Median	Range	*P* value
BRVO	2.00	1.10–2.60	0.120
CRVO	2.30	1.10–2.60	
PCV	2.30	2.00–3.00	
PDR	2.30	1.90–2.60	

**Table 4:** Comparison of postoperative BCVA among the four major etiological groups.

BCVA	Median	Range	*P* value
BRVO (G1)	1.00	0.50–2.00	0.001^*∗*^
CRVO (G2)	2.00	1.00–2.30	
PCV (G3)	2.00	1.70–2.60	
PDR (G4)	2.00	1.40–2.30	

G, group. ^*∗*^: G1 vs. G2: 0.047; G2 vs. G3: 1.000; G1 vs. G3: 0.001; G3 vs. G4: 1.000; G2 vs. G4: 1.000; G1 vs. G4: 0.010.

**Table 5:** Comparison of the final BCVA between the two groups with different initial BCVAs.

Final BCVA	*N* (%)	Median	Range	*P* value
GA (BCVA ≤ HM)	25 (56.82)	2.00	0.60–2.60	0.001
GB (BCVA > HM)	19 (43.18)	1.10	0.50–2.60	

G: group; *N*: number of eyes.

## Data Availability

The data used to support the study are included in the paper and further inquiries can be directed to the corresponding author/s.
